# Proceedings of the Twenty-First Annual Meeting of the Northern Ohio Dental Association

**Published:** 1880-06

**Authors:** L. C. Kelsey


### PROCEEDINGS
### OF THE
### TWENTY - FIRST ANNUAL MEETING
### OF THE
### Northern Ohio Dental Association.

**Held in Akron, Ohio, May 11th & 12th, 1880.**

The meeting was called to order at 10½ o'clock A. M., May 11th, by the President, J. W. Lyder, of Akron.

The minutes of the last meeting of the Association were read by the Secretary and on motion approved.

Reports of officers and Standing Committees being in order: the Treasurer, J. E. Robinson, presented a financial report, showing a balance in the Treasury of the Association of $28.85.

On motion an Auditing Committee was appointed, consisting of Drs. W. P. Horton and Chas. Buffett.

The President then proceeded to fill some vacancies on the several committees, which made them stand as follows:

Examining Committee: Charles Buffett, J. F. Siddall and J. R. Bell.

Executive Committee: L. Buffett, W. P. Horton.

Committee on Ethics: J. E. Robinson, L. Buffett and C. R. Butler.

On motion all dentists present not members of the Association, were invited to take part in the discussions.

The Committee on Ethics brought before the Association the case of Dr. C. B. Knowlton who, at the last meeting of the Society was suspended for unprofessional conduct; Dr. Knowlton having made no apology or explanation for the course he had pursued towards his professional brethren, was on motion expelled from the Association.

The Auditing Committee reported that they had examined the accounts of the Treasurer and found them correct. On motion the report was adopted. The next order of business was

READING OF ESSAYS.

*Dr. J. F. Siddall* of Oberlin being called upon read a poem, interspercing it with some unique remarks: his theme was "The Present Status of Filling Teeth." His manner of handling the subject caused some amusement and called out expressions of approval.

The Examining Committee then being in waiting reported the name of A. J. Douds of Canton, O., as a suitable person to become a member of the Association. On motion Dr. A. J. Douds was unanimously elected to membership.

*Dr. W. P. Horton* read an article from a California Dental Journal entitled: "The Office Boy's Dental College," prefacing it with appropriate remarks, in which he said, that in very large communities there was a certain few dentists who were always sure of business from the best classes: of others, those who make the greatest display through circulars and newspaper advertisements, and offer the cheapest work control the patronage of a large class of the less intelligent, to the detriment of those in the profession, who are trying to do *standard* work. The main drift of the article and of Dr. Horton's remarks was to show that too many of the boys who flock into the dental offices, do so from a mistaken idea, that it is a quick and easy way to fortune: forgetting that the only road to success and true merit is from long and faithful study.

On motion the Association adjourned to meet at 2 o'clock P. M.

AFTERNOON SESSION, MAY 11th.

The meeting was called to order by the President J. W. Lyder.

The minutes of the morning session were read and approved.

The Executive Committee presented the following subjects for discussion:

1st.—Present Status of Filling Teeth.

2nd.—Periodontitis: Cause, Prognosis, & Treatment.

3rd.—Tumors of the Mouth.

4th.—Cases in Practice.

During the session clinics by Drs. Palmer and Butler.

*Dr. L. Buffett* opened the discussion on the first subject by saying that the mode of preserving teeth by filling like everything else, had its ups and downs; but in the aggregate has always been on an upward grade. As to the time when filling teeth was first done, it is a matter of uncertainty.

At one time the *standard* in dentistry was very low. The principal work of the dentist being the replacement of missing teeth by artificial ones; and the standing of the operator depending much upon his skill in this particular direction. At another time it was the *filling* of teeth. Now it depends not only upon the ability of the operator but the conditions of the cases he is called upon to treat. The first fillings, of any account, were made of gold. This above all others has stood the test of time.

Other materials have been introduced and have served a good purpose.

To say that nothing but gold should be used is an insult to every dentist. Every one must be his own judge as to the materials best adapted to the cases presented. The *main* thing is to act honestly and conscientiously with himself and his patients. Notwithstanding there has been advancement, there has, at times, been a downward tendency; but on the whole, the dental profession is to-day on a higher plane than ever before. I am greatly in favor of soft foils: too much malleting in the use of cohesive gold has done harm. I think gold may become crystalized by too much hammering.

We should be eclectic in our practice. The use of soft foil with cohesive foil is now being regarded with great favor.

*Dr. Wilson* stated, that he often used soft and cohesive gold foil in the same cavities. Would put in the soft foil first with hand-pressure, then with the mallet build up and finish with cohesive. Young practitioners and students would do better work with soft than with cohesive foil.

*Dr. Templeton* remarked, that he had sometimes used amalgam. The first he used was Lawrence's. The great objection to amalgam is that it will shrink and fall out in time. For a long time in his practice did not use any material for filling but tin and gold foils. Lately had used some of the oxychlorides. Consider

Pierce's oxy-phosphate the best. He regards gold foil as the *best* filling material and his preference was for Kearsing's unannealed. In the buccal cavities of the second lower molars generally use tin foil.

*Dr. Ensign* said as reference has been made to Kearsing's gold foil I wish to speak in favor of it. This is unannealed foil and comes to us just as it leaves the beaters; it is not handled. The less you handle foil the better. This gold you can anneal as you desire, and it always works easy and nice.

*Dr. King*: This I regard as a very important subject. One speaker refered to the use of the mallet, stating that excessive malleting deteriorated the gold. He thought that was a mistake. The principal reason why there is leakage about fillings is lack of skill in impacting the gold. If the gold is not throughly impacted from the begining as you build up and increase the force, it will be drawn away from the margins. In regard to amalgams: he said there was a property in them which must be overcome before they could be of much use; and that property is *contraction*. This is the curse of all amalgams. All leaking fillings of what ever kind are useless.

*Dr. Bell* remarked, that too much malleting was harmful. Had seen teeth filled by the use of the electric mallet where the walls and especially the margins of the cavity seemed to be pulverized.

*Dr. Templeton* said I do not like the electro-magnetic mallet, it cuts the gold, destroys its wielding properties and disintegrates the walls of the cavities. It strikes so fast, that one vibration does not cease before another is offered. Much skill and practice is required to use it with any degree of success.

In regard to amalgams: think that the gold and platinum alloy about the poorest. In mixing this alloy the platinum will not unite with mercury. In some amalgams there is copper, which comes from the coin used in making it: and this is a detriment to amalgams.

*Dr. Douds* stated that when he commenced practice twenty years ago, he used tin and soft gold foil. Since that time many new things have come up from year to year, and he had made use of nearly all hoping to get the right material at last, but nothing as yet had been presented equal to tin and gold foils.

Amalgam fillings are generally defective. As much care should be taken in making tin filling as any. Had seen good tin fillings of twenty years standing.

*Dr. D. Gibbons* remarked, that amalgam as a filling material should not be made prominent in our discussion of this subject. We need to discuss this topic in such a manner as to encourage and aid the younger members of the profession, and show to them that we are on a higher plane than ever before.

*Dr. Erwin* said, in preparing the cavity for a gold filling I generally cut a grove instead of forming retaining pits. The main thing is to do the work throughly: In putting in a filling the instrument should not be allowed to touch the enamel. Sometimes I use only hand pressure and sometimes the mallet.

*Dr. Horton*: I generally use two kinds of foil in the same filling. My foil is prepared by rolling and then cutting into strips about ¼ of an inch in length. I first use soft foil then cohesive to build up and finish.

When I use amalgam I finish the filling by throughly burnishing it over with heavy tin foil, this absorbs the surplus mercury and leaves a nice surface.

*Dr. Poole*: Would like to ask if the tubuli of the dentine would ever leak so as to discolor?

*Dr. Robinson*: I am of the opinon leakage is always around the margin of the cavity and no where else.

*Dr. Butler*: I would not attempt to answer the question of Dr. Poole, but I cannot conceive of any other leakage than that around the margins of the filling. Much has been said pro and con: and I fear we have drifted away from the real intent of the subject: The *present* status of filling teeth; Has there been any advance in this direction? From much that has been said, one might think that no progress had been made. I am of the opinon there has been great progress. No one would say that he put in as good fillings when he first commenced practice as he now can after ten or fifteen years experience. One of short and limited practice cannot expect to have attained to as much skill as one of large and varied experience. From the fact of so good and reliable men coming into the profession is an evidence that we have made some progress.

The demand of the people is for good and honest work by men of integrity. I do not want to see the status of any one resting upon what a few say and do. Would like to see more independence of thought and action.

As reference has been made to the Electric Mallet, in condensing gold, I would like to say a few words in regard to its use. I cannot see how any one can use it with any certainty as to the result. There is a tremble or vibratory motion in using it which must be injurious. It will cut or break up the crystals of gold: and by the rapidity of its strokes injure the margins before one is aware of it. When we have come to the conclusion that we have made some failures, then there are signs of progress. Had we a free and honest expression from the profession in the United States to-day we would find that they are using less cohesive gold and less force in filling teeth than formerly. We have a different class to treat than we had fifteen or twenty years ago and we are up to the demand.

*Dr. Horton*: I have grown in my knowledge of filling teeth; and think I am still advancing. Owing to the financial condition of the country for a few years past there was a demand for cheap work, hence a class sprang up to fill the demand, and by extensive advertising secured patronage. It is an easy matter to get into the newspapers and buy up editorials: and it is humilating to see how the press will sometimes pamper to the interest of some, when well paid for it, regardless of the general good. In this way the status of filling teeth has been lowered. But this condition of things is passing away: we are getting in a healthier state, there is a greater demand for better work. From my observation I can see an upward tendency. The public is now more disposed to patronize skilled labor than in years past. I think it is not a question now as to how much per hour or what price for operations, but rather where can the best work be obtained. In regard to this association I think its status far ahead of what it was one or two years ago.

*Pres. Lyder* said that in the profession there had always been two classes, one of quacks, the other of honest faithful operators. The standing of the profession to-day shows less quackery than ever before. More is demanded of a dentist and more and better he is able to do. Our modes, appliances and instruments are better and the result is a higher status. What we now need is a little more independence of thought, *more energy*, more vim.

*Dr. C. Buffett*: We have now been over a great deal of ground and the whole matter resolves itself into this, the quality of the operation; no doubt it is better than years ago. Formerly there was too much copying of others: now there is more self reliance, more inquiry. I would say here one word in regard to the instructions in some of our dental colleges. It has been said that they teach nothing in reference to the use of amalgam. Now, as it has been, is now, and is to be, a filling material; it would seem proper that students in these colleges should be instructed *when* and *how* to use it. Until amalgam is wholly ignored, young practitioners should understand when its use is demanded. The independence of thought in the profession is an evidence of advancement.

On motion the Association adjourned to meet again at 7½ o'clock in the evening.

---

TUESDAY EVENING SESSION, MAY 11th.

Meeting called to order at 7½ o'clock, by the President.

The second subject; Periodontitis: Causes, Prognosis and Treatment, was taken up.

The discussion was opened by *Dr. L. Buffett*, who said that the subject of the inflammation of the investing membrane of the tooth is one of the most difficult we have to contend with. The causes producing periodontitis are various. It may result from local causes or from constitutional peculiarities. Ordinarily it is the result of a dead or dying pulp. We have decay of the tooth structure first, causing irritation of the pulp, then inflammation extending to the apex of the tooth: gases are then formed and the result is periodontitis. Sometimes the accumulation of tartar may cause it. The tendency to, and the severity of periodontitis is often modified by the constitutional peculiarities of different individuals.

Chiefly what we have to contend with results from local causes or injuries. Periodontitis often results in alveolar abscess. Our aim should be to avoid such a condition. Besides local injuries there may be some hidden causes; such as blood poison or syphilitic taint, tending to produce periodontitis. No *positive* or uniform treatment can be given in all cases. As a rule remove the cause and nature will do her work. Sometimes extraction is the only thing indicated. When there is much congestion, depletion is good treatment. The use of sulp. of Morphia injected into the inflamed parts will often give immediate relief.

*Prof. Watling* of Michigan University being called upon, related a very interesting case of periodontitis, where the congested condition of the pulp was seen through the walls of the tooth by the use of a powerful magnifying glass. In this case he effected a cure by the application of leeches.

*Dr. Buffett*: When the pulp is in a state of great congestion, what do you think of the probabilities of saving the tooth alive?

*Prof. Watling*: The constitution and habits of the patient being favorable, a tooth in that condition may often be saved alive by depletion.

*Dr. Buffett*: I think we would scarcely look for this result from local treatment only.

*Dr. Horton*: Very many good suggestions have been given us by Dr. Buffett, and we would do well to profit by them. No doubt there are many causes tending to produce periodontitis. It may result from nervous prostration, grief, blood poison, syphilis, &c. Hence no one line of treatment can be marked out for all cases. Ascertain the cause and treat accordingly. We are apt to think that the office of the dentist is to treat the teeth locally; but we should be able to treat our patients constitutionally as well.

*Dr. Gibbons*: I think we cannot, when we have much practice, find out every thing relative to the causes and treatment needed of many of the cases placed in our hands. We can treat locally, but to treat systemically, would seem to be out of the usual line of practice.

It is not expected of us. I have used the lancet with good results in treating periodontitis.

*Dr. Ensign*: We are not all able to treat these cases as they should be treated. We are not as a general thing prepared to treat periodontitis otherwise than by local remedies. Our knowledge is such, that we dare not venture on a course of systemic treatment; and this is humiliating.

*Dr. King*: I believe that periodontitis may sometimes be caused by attrition or by a blow on the tooth. But decay is more often the cause of the inflammation of the pulp. In the incipient stages of inflammation of the pulp the decay may be carefully removed and by proper treatment the tooth saved alive. And even when there is considerable congestion of the pulp, the tooth may sometimes be saved.

*Dr. Bell*: This is to me the most interesting subject on our programme.

Hard biting or a blow upon the tooth may cause periodontitis. The treatment depends upon the condition of the patient and the parts affected. In most cases I would be opposed to the use of the lancet. I have used aromatic sulphuric acid with good results.

*Dr. Butler*: It has been said that the periosteum after having taken on this inflammatory action, is never again restored to its normal condition. I believe this is true: the membrane will be more or less thickened ever after. Sometimes irritating or working the tooth may set up an inflammatory action, which would result favorably.

*Dr. Palmer*: In incipient stages of periodontitis manipulating the gums with the fingers, is good practice; and may effect an entire cure.

*Dr. Siddall*: In treating periodontitis, I often make an opening through the gums to the apex of the tooth and apply carbolic acid.

*Dr. Palmer*: In most cases I would treat through the root, making a free opening to the apex.

On motion the Association adjourned to meet again at 8 o'clock in the Morning for Clinics.

WEDNESDAY MORNING, 8 O'CLOCK, MAY 12th.

---

Clinics were held in the office of Dr. J. W. Lyder, at which time Dr. C. Palmer showed to the members of the profession how to apply the rubber dam in difficult cases. He also exhibited clamps of his own making, explaining their advantage over others.

Drs. C. R. Butler and J. W. Lyder gave clinics on filling teeth, which consumed the time until 11 o'clock, when the Association went into session.

The Examining Committee reported the names of Drs. D. E. Fenn, W. A. Porter and D. Gale French as suitable persons to become members of the Association ; and on motion they were elected to membership.

The Association then proceeded to the election of officers for the ensuing year, the result of which was as follows :

For President—*Dr. Charles Buffett of Cleveland, O.*
" Vice-President—*Dr. J. G. Templeton of Pittsburg, Pa.*
" Cor. Secretary—*Dr. H. F. Harvey of Cleveland, O.*
" Rec. Secretary—*Dr. L. C. Kelsey of Elyra, O.*
" Treasurer—*Dr. J. E. Robinson of Cleveland, O.*

The newly elected President was then conducted to the chair by Drs. Jennings and Butler. The retiring President, J. W. Lyder, made a few very appropriate remarks, thanking the Association for the honor conferred upon him in having been twice elected as their President. He spoke encouragingly of the interest and general good feeling, which had prevailed in the Association for the three years, he had presided over it. A motion was made and carried, that when the Association adjourns, it shall be to hold its next annual meeting in Cleveland, Ohio, on the second Tuesday and Wednesday of May 1881.

Delegates selected to attend the American Dental Association, to be held in Boston, Mass., in August next, as follows : Drs. I. H. Brown, C. Palmer, H. L. Ambler, J. W. Lyder, G. H. Wilson and D. Gale French. On motion the Association adjourned to meet at 2 o'clock P. M.

———

AFTERNOON SESSION, MAY 12th, 1880.

Association called to order at 2 o'clock P. M. President C. Buffett in the chair. The following committees were filled by appointment of the President.

Examining Committee, Drs. J. W. Lyder, D. R. Jennings and J. R. Bell.

Executive Committee, Drs. W. P. Horton and E. W. Poole.

On motion the Second subject was passed and the Third one taken up for discussion, viz, Tumors of the mouth.

*Dr. Templeton* said that the general impression is that Dentists have no right to treat tumors of the mouth. I think this impression should be removed.

Dentists should be able to diagnose tumors in the mouth and treat them upon the best known principles in medicine and surgery. This I think should come under the head of specialties in dental practice. A dentist should have knowledge enough of physology, anatomy and surgery to give him confidence in treating any of the tumors of the mouth.

*Dr. Horton*: It is a difficult matter to handle this subject unless one has made it a special study. We are too apt to ignore this branch of dentistry, and refer cases of tumors in the mouth to the surgeon.

The Doctor then related several cases which occured in his own practice.

*Dr. Butler* then made a motion that this subject be passed and presented again at the next annual meeting of the Association—carried.

The last subject. Cases in practice was then taken up, but owing to the lateness of the hour, very few were presented.

*Dr. Lyder* spoke of removing a tumor from the mouth of one of his patients with the most satisfactory results.

*Prof. Watling* spoke of replanting teeth, and related a few cases of failure by absorption of the roots.

Cases were also presented of tumors in the mouth, treated by Dr. Buffett which were very interesting.

*Dr. C. Palmer* gave a very interesting talk as to his manner of putting crowns upon roots, and of building up contoures.

Time and space will not allow me to report in detail his valuable remarks.

On motion the Association adjourned to meet in Cleveland, Ohio, on the Second Tuesday and Wednesday of May, 1881.

L. C. KELSEY, Rec. Secretary.

